# Generation of a Transplantable Population of Human iPSC-Derived Retinal Ganglion Cells

**DOI:** 10.3389/fcell.2020.585675

**Published:** 2020-10-27

**Authors:** Oriane Rabesandratana, Antoine Chaffiol, Antoine Mialot, Amélie Slembrouck-Brec, Corentin Joffrois, Céline Nanteau, Amélie Rodrigues, Giuliana Gagliardi, Sacha Reichman, José-Alain Sahel, Alain Chédotal, Jens Duebel, Olivier Goureau, Gael Orieux

**Affiliations:** ^1^Institut de la Vision, Sorbonne Université, INSERM, CNRS, Paris, France; ^2^CHNO des Quinze-Vingts, INSERM-DHOS CIC 1423, Paris, France; ^3^Department of Ophthalmology, The University of Pittsburgh School of Medicine, Pittsburgh, PA, United States

**Keywords:** retinal ganglion cells, induced pluripotent stem cells, retinal organoids, cell transplantation, optic nerve injury

## Abstract

Optic neuropathies are a major cause of visual impairment due to retinal ganglion cell (RGC) degeneration. Human induced-pluripotent stem cells (iPSCs) represent a powerful tool for studying both human RGC development and RGC-related pathological mechanisms. Because RGC loss can be massive before the diagnosis of visual impairment, cell replacement is one of the most encouraging strategies. The present work describes the generation of functional RGCs from iPSCs based on innovative 3D/2D stepwise differentiation protocol. We demonstrate that targeting the cell surface marker THY1 is an effective strategy to select transplantable RGCs. By generating a fluorescent GFP reporter iPSC line to follow transplanted cells, we provide evidence that THY1-positive RGCs injected into the vitreous of mice with optic neuropathy can survive up to 1 month, intermingled with the host RGC layer. These data support the usefulness of iPSC-derived RGC exploration as a potential future therapeutic strategy for optic nerve regeneration.

## Introduction

Retinal ganglion cells (RGCs) play a major role in the visual function in transmitting visual information from the retina through the optic nerve and optic tract to the brain structures dedicated to processing visual information. RGC impairment is a common feature in many pathologies leading to visual loss, generally referred to as optic neuropathies such as Leber’s hereditary optic neuropathy (LHON), and dominant optic atrophy or glaucoma, which is the second cause of blindness in the world (Jonas et al., 2017). Elevated intraocular pressure (IOP) is considered an important risk factor for glaucoma and most of the treatment strategies aim at reducing IOP. However, despite treatments, RGC degeneration and visual loss still progress in some patients leading to irreversible blindness (Susanna et al., 2015; Greco et al., 2016). Moreover, some patients are diagnosed at a late stage of the disease when many RGCs have already been lost. In hereditary optic neuropathies, RGC loss mainly occurs in young patients, notably LHON-patients with no effective treatment to date (Newman, 2012; Carelli et al., 2017).

Human RGCs are difficult to access and grow *in vitro* (Zhang et al., 2010). Therefore, human pluripotent stem cells (hPSCs) represent one of the most promising sources of human RGCs. Recent development of methods guiding the differentiation of hPSCs toward specific retinal lineages, including RGCs, has emerged as a powerful strategy for disease modeling, drug screening, and gene or cell therapy (Llonch et al., 2018; Rabesandratana et al., 2018; Miltner and La Torre, 2019; Ahmad et al., 2020). Previous studies have demonstrated the ability to differentiate RGCs from plated hPSC-derived embryoid bodies (Riazifar et al., 2014; Sluch et al., 2015; Gill et al., 2016; Teotia et al., 2017). Based on initial protocols developed with mouse and human ESCs (Eiraku et al., 2011; Nakano et al., 2012), different groups including ours developed three-dimensional (3D) culture systems recapitulating key steps of retinal development and allowing the generation of self-organizing retinal organoids containing RGCs (Reichman et al., 2014; Zhong et al., 2014; Maekawa et al., 2015; Ohlemacher et al., 2016; Fligor et al., 2018). Very recently, RGCs were differentiated from human induced pluripotent stem cells (hiPSCs) using a chemically defined medium resulting in dual SMAD and Wnt inhibition bypassing retinal organoid formation (Chavali et al., 2020). Patient-specific iPSCs can be useful to better characterize the pathogenesis and molecular mechanisms of different inherited optic neuropathies (Chen et al., 2016; Ohlemacher et al., 2016; Wu et al., 2018; VanderWall et al., 2020). iPSC-derived RGCs also offer opportunities to identify molecules with therapeutic potential (Chen et al., 2016; Sluch et al., 2017) or to evaluate the efficiency of rescue strategies (Hung et al., 2016; Wong et al., 2017). Finally, hPSC-derived RGCs could be used for cell therapy even if many obstacles need to be overcome before any clinical application, such as the refractory nature of the central nervous system to axonal regeneration that could impede the reconnection of new RGC axons to their visual targets (Fischer et al., 2017; Laha et al., 2017). The ability to purify hPSC-derived RGCs from other cell types and to eliminate any residual proliferative cells is also a critical point to obtain a population of transplantable cells. Genetic engineering has been used to facilitate RGC isolation employing RGC-specific reporter gene or RGC-specific cell surface marker (Sluch et al., 2015; Kobayashi et al., 2018).

Based on our good manufacturing practice (GMP)-compliant retinal differentiation protocol (Reichman et al., 2017), we demonstrate that RGCs cultured in 2D conditions after dissociation of early retinal organoids derived from hiPSCs strongly express the cell surface antigen THY1 (also known as CD90). Here, we report a molecular and functional characterization of iPSC-derived RGCs and demonstrate the ability to enrich the RGC population using a THY1-based magnetic-activated cell sorting (MACS) strategy. Transplantation of enriched THY1-positive RGCs derived from a new fluorescent GFP reporter iPSC line in a mouse model of RGC degeneration supports the convenience of our culture and selection strategy when studying the potential of hPSC-derived RGCs for cell therapy for optic neuropathies.

## Materials and Methods

### Animals

Eleven to 13-week-old adult female C57/BL6J mice were used in this study (Envigo). Animals were kept on a 12-h light/12-h dark cycle and allowed to eat and drink *ad libitum* (certified animal facility of the “Institut de la Vision”; agreement number A751202). All experiments were carried out in strict accordance with the Association for Research in Vision and Ophthalmology statement for animal research in ophthalmology. Moreover, all protocols were approved by the local ethical committee (Charles Darwin Ethical Committee for Animal Experimentation C2EA-05) in strict accordance with French and European regulation for animal use in research (authorization number #9061).

### Human Induced Pluripotent Stem Cell Cultures

Two established human iPSC lines, hiPSC-2 and hiPSC line-5f, derived, respectively, from dermal fibroblasts (Reichman et al., 2014) and retinal Müller glial cells (Slembrouck-Brec et al., 2019) were cultured as previously described (Reichman et al., 2017). The fluorescent reporter iPSC line adeno-associated virus integration site 1 (AAVS1):CrxP_H2BmCherry-hiPSC line (Gagliardi et al., 2018) has also been used for specific experiments. Briefly, hiPSC lines were cultured on feeder-free conditions on truncated recombinant human vitronectin rhVTN-N (Thermo Fisher Scientific) in Essential 8^TM^ medium (Thermo Fisher Scientific). Cells were routinely cultured at 37°C in a standard 5% CO_2_/95% air incubator with a daily medium change.

### Generation of Human Reporter AAVS1::CAG-P_EGFP hiPSC Line

We used pX330-U6-Chimeric_BB-CBh-hSpCas9 (addgene#42230) (Cong et al., 2013) to construct the CRISPR/Cas9 vector by annealing and ligating in *Bbs*I sites an oligonucleotide pairs (5′-CACCGGGGCCACTAGGGACAG GAT-3′/3′-CCCCGGTGATCCCTGTCCTACAAA-5′) encoding a 20-nt AAVS1 guide sequence according to the protocol of Ran et al. (2013). We used the AAV–CAGGS–EGFP plasmid (Addgene #22212) (Hockemeyer et al., 2009) containing Puromycin-resistant gene and EGFP under the control of CMV early enhancer/chicken β actin (CAG) promoter between AAVS1 homology arms of 800 bp each (HA-L and HA-R). Plasmids are prepared using a Plasmid Midiprep kit (Macherey–Nagel) and verified for identity and integrity by restriction digest and gel electrophoresis before storage at 1–2 mg/ml in sterile Tris buffer (pH 8.0). The generation of stable cell clones was performed as previously described (Gagliardi et al., 2018). The hiPSC line-5f clone was grown for 24 h in six-well plates before overnight transfection at 37°C with CRISPR/Cas9 and donor plasmids using FuGene HD (ratio 1:1:5; Promega). Two days after transfection, selection of recombinant cells was started in the presence of 0.25 μg/ml puromycin (Thermo Fisher Scientific) for the first 48 h; the concentration of puromycin was then increased to 0.5 μg/ml until picking of single colonies. Genomic DNA from puromycin-resistant clones was extracted with NucleoSpin Tissue kit (Macherey–Nagel) according to the manufacturer instruction. Correct reporter integration was evaluated by PCR using a forward primer upstream to the left arm of AAVS1 of recombination and a reverse primer either within the reporter cassette or in the AAVS1 right homologous arm. The PCR was composed of 32 cycles including three successive steps at 95°C, 60°C, and 72°C, respectively, for 30 s, 30 s, and 2 min. The genotype was finally visualized after migration of the DNA in a 1.2% agarose gel. After validation by sequencing, one homozygous reporter integration cell line was selected, and pluripotent status was validated by immunostaining for pluripotency markers, and the integrity of the karyotype was verified by SNP genotyping using Illumina’s Infinium HumanCore-24 Bead Chips (Illumina, Inc., San Diego, CA, United States) at Integragen (Évry, France) (Slembrouck-Brec et al., 2019).

### Retinal Differentiation

Retinal differentiation was based on our previously established protocol with adherent human iPSCs (Reichman et al., 2017; Slembrouck-Brec et al., 2019). After 56 days of differentiation (D56), retinal organoids were collected and enzymatically dissociated with papain (Worthington, WOLS03126) as previously described (Reichman et al., 2017). Retinal organoids were incubated with preactivated papain (1 U for 10^6^cells) in Ringer solution during 30 min at 37°C, and then gently pipetted up and down to obtain uniform cell suspension. Dissociated retinal cell suspension was plated at a density of 100,000 cells/cm^2^ onto either 6-, 24- (Corning, 3528), 48- (Greiner bio-one), or 96-well plates (Cellvis) previously coated with poly-D-lysine (Sigma-Aldrich) and laminin (Sigma-Aldrich), respectively, at 2 and 1 μg/cm^2^. Dissociated retinal cells were cultured in retinal differentiation medium (RDM) composed of DMEM/F12 plus B27 supplement (Reichman et al., 2017), with medium changed every 2–3 days for 1 week after plating.

### Cryopreservation of Retinal Organoids

Up to 12 retinal organoids at D45 were suspended in 250 μl of cold CryoStem freezing medium (CliniSciences) and frozen in a 1.5-ml cryogenic tube (Sarstedt) placed in isopropanol-based Mr. Frosty freezing container (Thermo Fisher Scientific) at −80°C for a minimum of 4 h. Frozen tubes were kept in a −150°C freezer for long-term storage. Frozen retinal structures were thawed quickly at 37°C in a water bath and resuspended in pre-warmed dedicated media for downstream investigations.

### Magnetic-Activated Cell Sorting

One week after enzymatic dissociation at D56, dissociated retinal cells were carefully detached from the plates by incubation with previously activated papain protocol (1 U for 10^6^ cells) for 15 min at 37°C. To remove residual aggregates, deoxyribonuclease I from bovine pancreas (Sigma-Aldrich) was added into cell suspensions, before filtering through a 30-mm strainer (Miltenyi Biotec) prehydrated with 1 ml of RDM. Cell suspension was centrifuged for 10 min at 300 *g* and resuspended in MACS buffer (80 μl up to 2.10^7^ cells) for 30 min at 4°C to block non-specific binding sites. Next, cells were incubated with anti-mouse IgG1 MicroBeads (Miltenyi Biotec) coupled with human THY1 antibody for 15 min at 4°C at a dilution of 1:5 for a ratio up to 2.10^7^ cells. The cells were washed by adding 2 ml of MACS buffer, centrifuged at 10 min at 300 × *g* and resuspended in 500 μl of MACS buffer (MACS BSA solution) supplemented with 1:20 MACS separation buffer (Miltenyi Biotec). Cell suspension was applied once onto a pre-equilibrated MS column (Miltenyi Biotec) fixed to a MiniMACS^TM^ separator (Miltenyi Biotec). The flow-through containing unlabeled cells was collected and then applied a second time onto the separation column to optimize the selection. The column was washed twice with 500 μl of MACS buffer. The positive fraction was eluted with 1 ml of MACS buffer following removal of the column from the magnet.

### Flow Cytometry

After THY1 MACS, all unsorted and sorted THY1 retinal cell fractions were resuspended to a final volume of 1 ml in MACS buffer. Then they were incubated with FITC-conjugated THY1 primary antibody for at least 30 min, at 4°C, in the dark. To exclude dead cells, propidium iodide was added to the cells at a final concentration of 1 μg/ml, incubated for 10 min at 4°C in the dark. Background fluorescence and non-specific binding were measured using unstained cells and mouse IgG1 FITC-conjugated isotypic antibody control (R&D systems) with a concentration of 5 μl/test up to 10^6^ cells. Samples were analyzed without additional washings. Analysis was performed with FC500 Flow Cytometer (Beckman Coulter). Flow cytometry data were analyzed using FlowJo software (TreeStar). A minimum of four independent biological experiments were performed for flow cytometry.

### Multi-Electrode Array Recordings

Whole retinal organoids were chopped at D56 and plated onto a multi-electrode array (MEA) chip (MEA256 100/30 iR-ITO, Multi Channel Systems, Germany) facing the electrodes and coated with poly-D-lysine/laminin to promote adhesion onto the electrodes. Organoid fragments were maintained during 2–3 weeks in RDM at 37°C in a standard 5% CO_2_/95% air incubator, with medium changed every 2–3 days until electrophysiological recordings. During MEA recordings, the organoids were continuously perfused with oxygenized (95% O_2_, 5% CO_2_) Ames medium (Sigma-Aldrich) at 34°C at a rate of 1–2 ml/min (Garita-Hernandez et al., 2019). Raw extracellular RGC activity was amplified and sampled at 20 kHz. Resulting data was stored and filtered with a 200-Hz high-pass filter for subsequent offline analysis using Spike2 software v.7 (CED Co., United Kingdom). Single-unit data were obtained using a combination of template matching and cluster grouping based on principal component analysis of the waveforms. Each recording lasted for at least 30 min.

### Single-Cell Electrophysiological Recordings

Patch-clamp electrodes were made from borosilicate glass (BF100-50-10, Sutter Instruments) and pulled to 6–9 MΩ. Pipettes were filled with 115 mM K Gluconate, 10 mM KCl, 1 mM MgCl_2_, 0.5 mM CaCl_2_, 1.5 mM EGTA, 10 mM HEPES, and 4 mM ATP–Na_2_ (pH 7.2). An Axon MultiClamp 700B amplifier was used for whole-cell patch-clamp and extracellular recordings. Retinal cells were recorded 2–3 weeks after plating, either in voltage-clamp configuration and clamped at −60 mV (with 10 mV steps from −100 to +40 mV), or in current-clamp configuration (with current injections from −20 to +100 pA). Extracellular recordings were also performed, with electrodes filled with Ames’ solution. During recordings, cells were perfused with oxygenized (95% O_2_, 5% CO_2_) Ames’ medium (Sigma-Aldrich) at 34°C at a rate of 1.5 ml/min.

### RNA Extractions and TaqMan Assays

Total RNAs were extracted using NucleoSpin RNA XS kit (Macherey–Nagel) according to the manufacturer’s protocol, and RNA yields and quality were checked with a NanoDrop spectrophotometer. cDNA was synthesized from 250 ng of mRNA using the QuantiTect reverse transcription kit (Qiagen, 205313) following manufacturer’s recommendations. Synthesized cDNA was then diluted at 1/20 in DNase-free water before performing quantitative PCR. qPCR analysis was performed on an Applied Biosystems Real-Time PCR machine (7500 Fast System) with custom TaqMan® Array 96-Well Fast plates (Thermo Fischer Scientific) and TaqMan® Gene expression Master Mix (Thermo Fischer Scientific) following manufacturer’s instructions. All primers and MGB probes labeled with FAM^TM^ (carboxyfluorescein) for amplification (Supplementary Table S1) were purchased from Thermo Fischer Scientific. Results were normalized against 18S, and quantification of gene expression was based on the Delta Ct Method in three minimum independent biological experiments.

### Tissue Fixation and Cryosection

For eye cup collecting, animals were deeply anesthetized by intraperitoneal injection of ketamine (Ketamidor; Axience; 100 mg/kg) and xylazine (Nerfasin® Vet; Axience; 10 mg/kg) before transcardiac perfusion with 100 ml of 4% paraformaldehyde at 4°C. Retinal organoids and mouse eye cups were fixed (or postfixed) for 20 min in 4% paraformaldehyde at 4°C and washed in PBS. Structures were incubated at 4°C in PBS/30% sucrose solution for at least 2 h. Structures were embedded in a solution of PBS/7.5% gelatine/10% sucrose and frozen in isopentane at −55°C. For further analysis, 10- and 14-μm-thick cryosections were collected, respectively, for retinal organoids and mouse eye cups. Dissociated retinal cells on coverslips (WPI) or on glass-bottomed 96-well plates (Cellvis) were fixed with 4% paraformaldehyde for 15 min before immunostaining.

### Immunostaining, Imaging, and Quantification

After washes with PBS, non-specific binding sites were blocked for 1 h at room temperature with a PBS solution containing 0.2% gelatine and 0.1% Triton X-100 (blocking buffer) and then overnight at 4°C with the primary antibody (Supplementary Table S2) diluted in blocking buffer. Slides were washed three times in PBS with 0.1% Tween and then incubated for 1 h at room temperature with appropriate secondary antibodies conjugated with either Alexa Fluor 488, 594, or 647 (Supplementary Table S2) diluted at 1:800 in blocking buffer with 4′,6-diamidino-2-phenylindole (DAPI) diluted at 1:1,000 to counterstain the nuclei. Specifically, for mouse eye cups, sections were incubated in two drops of Mouse IgG blocking reagent (Vector laboratories) diluted in 2.5 ml of PBS for 1 h at room temperature to block endogenous mouse antibody in the tissue section in anticipation of mouse antibody- based immunostaining.

Cell death was detected using the *In Situ* Cell Death Detection kit, TMR (Roche), according the to the manufacturer’s instruction.

Fluorescent staining signals were captured with an Olympus FV1000. Acquisitions were done with a variable step size according to objective magnification (1.64 μm at ×20; 0.61 μm at ×40; 0.48 μm at ×60). Images were analyzed with FIJI/ImageJ software, and each illustration corresponds to a Z-projection of all X-Y optical sections.

Quantification of cell density immunoreactive for specific markers has been performed by automatic counting using ArrayScan VTI HCS Reader® station with the HCS iDev Cell® studio software 6.6.0. (Thermo Fischer Scientific). A minimum of 72 areas (0.83 mm^2^ each) were counted for each condition of dissociated retinal cells per experiment. For a few markers, manual quantification was realized on the FIJI/ImageJ software. Quantification is expressed as mean ± S.E.M and corresponds to a minimum of four independent biological experiments for automatic or manual counting.

### Optic Nerve Crush Mouse Model

Animals were anesthetized by intraperitoneal injection of ketamine (Ketamidor; Axience; 100 mg/kg) and xylazine (Nerfasin® Vet; Axience; 10 mg/kg). Buprenorphine (Buprecare, Axience; 0.05 mg/kg) was also administrated before the surgery to provide additional analgesia. Oxybuprocaine (THEA) was also administered locally on the operated eye. Under a stereoscopic microscope, the left eye ball was bulging with Dumont fine forceps (Fine Science Tools, n^o^ 11252-30) to expose the optic nerve. The optic nerve was crushed a few millimeters behind the eye by compression for 5 s with Dumont #5 – mirror finish forceps (Fine Science Tools, n^o^ 11252-23). Lubrital (Dechra) was used to avoid dry eyes until waking up.

### Transplantation of Human-iPSC-Derived RGCs

Mice were first anesthetized by 5% isoflurane (Isoflurin; Axience) inhalation for 2 min and were maintained in deep anesthesia with 2% isoflurane inhalation. Oxybuprocaine was also administered locally before transplantation. Cell suspension (1 μl) containing 200,000 cells in MACS buffer was delivered by intravitreal injection. Injection was performed using a micropump (UltraMicroPump III with Micro4 Controller; World Precision Instruments). Cells were delivered at 150 nl/s using a 10-μl NanoFil syringe with a 33G beveled tip (NanoFil). From 1 week before transplantation to the end of the follow-up, mice were maintained under immunosuppression through cyclosporine treatment (210 mg/l) in the drinking water. A total of 27 female mice were analyzed 1 week after transplantation (*n* = 17 animals with grafted THY1-positive cells; *n* = 4 animals with grafted unsorted cells) and 4 weeks after transplantation (*n* = 6 animals with grafted THY1-positive cells).

### Statistical Analysis

“n” corresponds to the number of organoids, animals, or images from each independent differentiation; “N” indicates the number of independent experiments performed (e.g., the number of independent retinal differentiations). All statistical analyses are based on at least three independent experiments. Data were averaged and expressed with means ± SEM. Statistical analysis was performed using Prism 7 (GraphPad software) with appropriate statistical tests including Mann–Whitney test, ordinary one-way, two-way ANOVA, or Kruskal–Wallis test followed when necessary by multiple comparison test (*post hoc* analysis) such as Dunn’s (multiple comparison with one control group) or Tukey’s test (comparison between all groups). *P* values lower than 0.05 led to the rejection of the null hypothesis (H0) and to consider the difference statistically significant.

## Results

### Absence of THY1 Expression in Human-Induced Pluripotent Stem Cell-Derived Retinal Organoids

**FIGURE 1:**
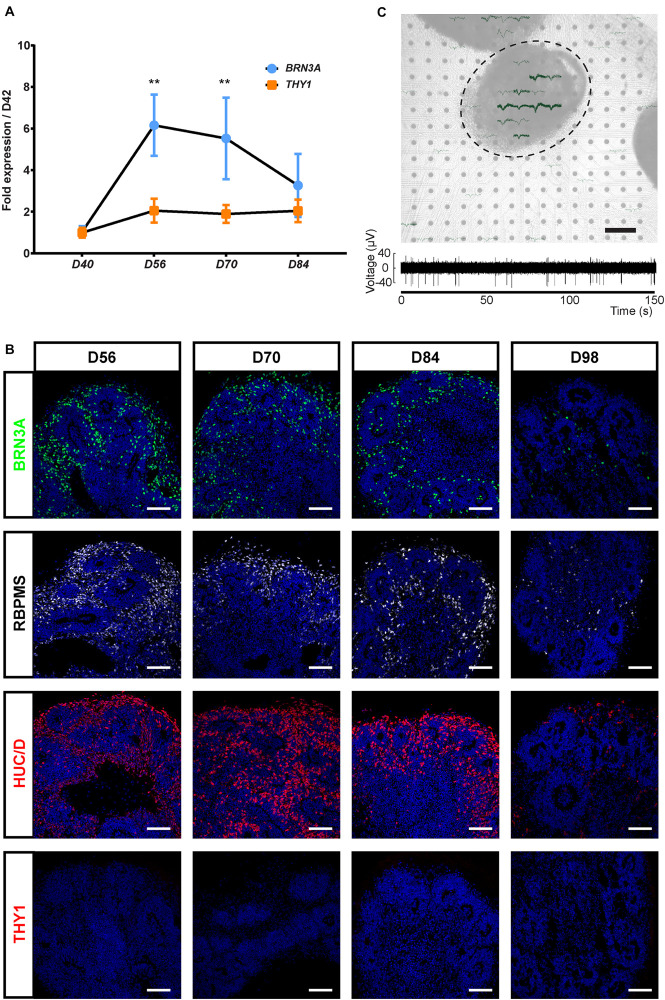
Characterization of the retinal ganglion cell (RGC) population in human induced pluripotent stem cell (hiPSC)-derived retinal organoids. **(A)** RT-qPCR analysis of *BRN3A* and *THY1* during differentiation between D42 and D84 (mean ± SEM; *N* = 3 differentiations per time point; *n* ≥ 10 organoids/differentiation). Gene expression at each time point is indicated relative to organoids at D42. **p < 0.01, Kruskal–Wallis test followed by Dunn’s multiple comparison. **(B)** Immunostaining showing the expression of BRN3A, RBPMS, HuC/D, and the absence of THY1 immunoreactivity in sections of retinal organoids, from D56 to D98. Nuclei were counterstained with DAPI (blue). Scale bars, 100 μm. **(C)** Top, infrared image displaying an organoid cultured on a 256-recording site (gray dots) MEA chipset. Electrical activity showed as superimposed waveforms is observed at the level of the organoid (*N* = 7 independent experiments including 10 electrodes showing electrical activity). Scale bar, 200 μm. Bottom, raw data trace example of a recorded neuron from the same organoid exhibiting spontaneous firing activity.

Because THY1 is considered as a specific marker of RGC in a retinal context (Barnstable and Dräger, 1984), we first sought to determine, using our previously GMP-compatible protocol (Reichman et al., 2017), the expression of this cell surface marker during the differentiation of hiPSC-derived retinal organoids in floating conditions. RT-qPCR analysis revealed a low increase in *THY1* expression in maturating organoids between D42 and D84 contrasting with the transient increase in *BRN3A* expression that demonstrated the generation of RGCs during this period (Figure 1A) (*n* ≥ 30 organoids from *N* ≥ 3 differentiations; ^∗∗^*p* < 0.01, Kruskal–Wallis test followed by Dunn’s multiple comparison). The transient expression of *BRN3A* can be explained by RGC loss in floating culture conditions (Reichman et al., 2014; Zhong et al., 2014; Capowski et al., 2019). Immunofluorescence analysis showed that numerous immunoreactive cells for specific RGC markers (BRN3A, RBPMS, and HuC/D) are present in floating retinal organoids at D56, D70, and D84, while their density decreased at D98 (Figure 1B) and became undetectable at later stages as previously described (Reichman et al., 2014). By contrast, the photoreceptor population was clearly traceable at D98 according to CRX and Recoverin (RCVRN) expression ruling out a global and non-specific cell loss (Supplementary Figure S1). This observation confirmed the data obtained by RT-qPCR and revealed the progressive RGC loss over long-term retinal organoid maturation. Surprisingly, immunofluorescence analysis did not detect expression of THY1 in retinal organoids between D42 and D98 (Figure 1B) despite the gene expression detected at mRNA level by RT-qPCR (Figure 1A).

In order to evaluate the functional maturation of presumed hiPSC-derived RGCs, and considering that retinal cells capable of emitting large spikes are overwhelmingly RGCs, we performed multi-electrode array (MEA) recordings from organoids cultured on MEA chipsets for 2 weeks (Figure 1C). Spiking activity was recorded at the surface of four out of seven MEA chipset, and the average spiking activity of individual cells was 0.37 ± 0.13 Hz (mean ± SEM; *N* = 4 out of seven experiments, *n* = 10 responding electrodes).

### Promotion of Human-iPSC-Derived RGC Survival and Maturation in Adherent Conditions

**FIGURE 2:**
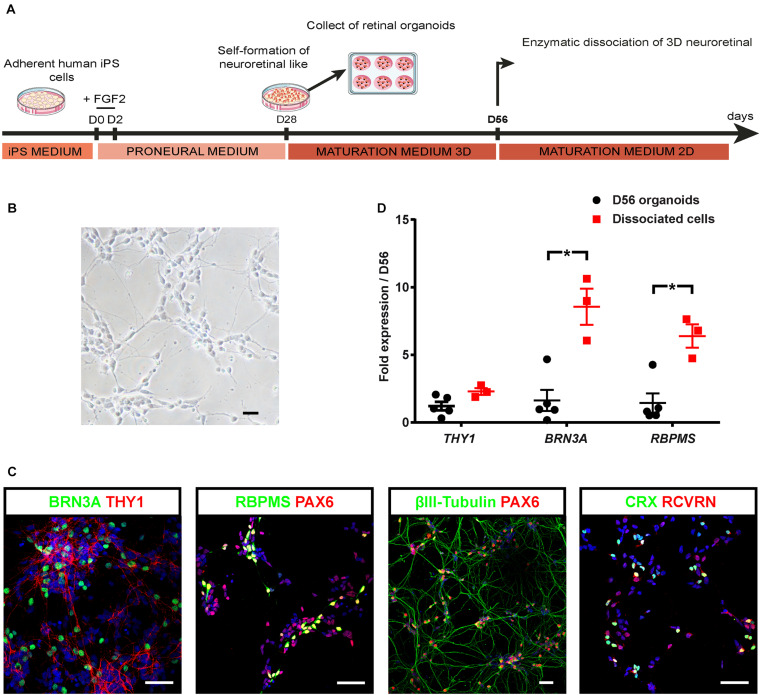
Optimization of hiPSC-derived RGCs differentiation. **(A)** Schematic diagram illustrating the protocol for RGC differentiation from hiPSC-derived retinal organoids. **(B)** Phase-contrast micrograph of retinal cells 1 week after plating of dissociated cells derived from D56 organoids; scale bar, 50 μm. **(C)** Immunostaining on dissociated cells derived from D56 retinal organoids, showing the co-expression of BRN3A and THY1, or PAX6 with RBPMS, or βIII-tubulin allowing the identification of RGCs 1 week after plating. Photoreceptors are also identified according to CRX and recoverin (RCVRN) expression. Nuclei staining with DAPI in blue; scale bars, 50 μm. **(D)** RT-qPCR analysis of *THY1*, *BRN3A*, and *RBPMS* in D56 organoids and in retinal cells 1 week after plating (mean ± SEM; *N* = 3 differentiations per time point; *n* ≥ 10 organoids/differentiations). Gene expression at each time point is indicated relative to D56 organoids. *p < 0.05, Mann–Whitney test).

Since targeting THY1 would be useful to isolate the RGC population, we aimed at inducing RGC maturation to trigger THY1 expression at protein level. For this purpose, we developed a two-stepwise retinal differentiation protocol to promote survival and maturation of RGCs (Figure 2A). Retinal organoids were dissociated after 8 weeks of differentiation (D56), and cell suspension was seeded back onto an adherent substrate for 1 week and maintained in RDM medium to enhance RGC survival and maturation. After 1 week, the dissociated retinal cells showed a typical neuronal morphology (Figure 2B). Most cells were identified as RGCs by the co-expression of BRN3A and THY1 (Figure 2C), or the co-expression of PAX6 with RBPMS or βIII-tubulin (Figure 2C; Fournier and McKerracher, 1997; Zhang et al., 2010; Rodriguez et al., 2014; Li et al., 2017). The co-expression of CRX and RCVRN in some cells confirmed that differentiating photoreceptors were also present (Figure 2C). RT-qPCR analysis revealed a higher expression of *BRN3A* and *RBPMS* in retinal cells 1 week after plating compared to D56 organoids (Figure 2D) (*n* ≥ 30 organoids from *N* ≥ 3 differentiations; ^∗^*p* < 0.05, Mann–Whitney test) and a weak but not statistically significant increase in *THY1* expression (Figure 2D) (*n* ≥ 30 organoids from *N* ≥ 3 differentiations). All these results confirmed an enrichment of maturating RGCs over other retinal cell populations in these adherent conditions.

**FIGURE 3:**
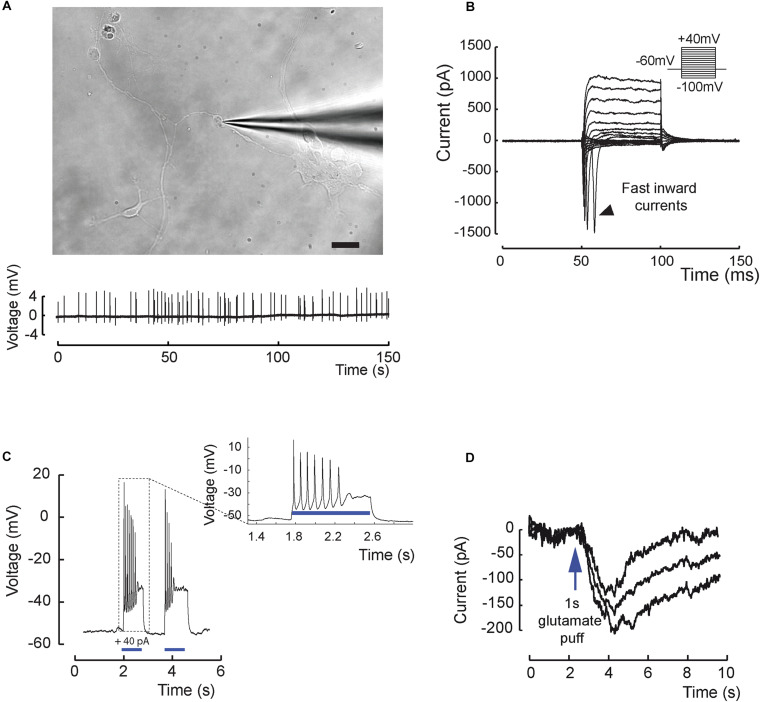
Excitability and electrical properties of hiPSC-derived RGC-like cells after differentiation in 2D. **(A)** Top, infrared image of a recording electrode in contact with a RGC 3 weeks after plating of retinal cells derived from D56 organoid Scale bar, 20 μm. Bottom, example of an extracellular recording from the same cell displaying spontaneous firing activity (0.31 ± 0.07 Hz; mean ± SEM; *N* = 9; *n* = 20 out of 61 recorded cells). **(B)** Whole-cell patch-clamp recording of a representative cell in voltage-clamp mode. Current responses to voltage steps are obtained by stepping the membrane potential from –100 to +40 mV in 10 mV increments. Note the presence of fast inward currents (arrow head); (*N* = 5; *n* = 15 out of 22 recorded cells). **(C)** Whole-cell patch-clamp recording in current-clamp mode. Injecting positive current (40 pA) in the cell (blue bars) leads to firing activity (*N* = 5; *n* = 7 out of 10 recorded cells). **(D)** Recording in one cell of a long inward current evoked by glutamic acid (1 mM) application and compatible with a synaptic current; this response has been reproduced three times.

**FIGURE 4:**
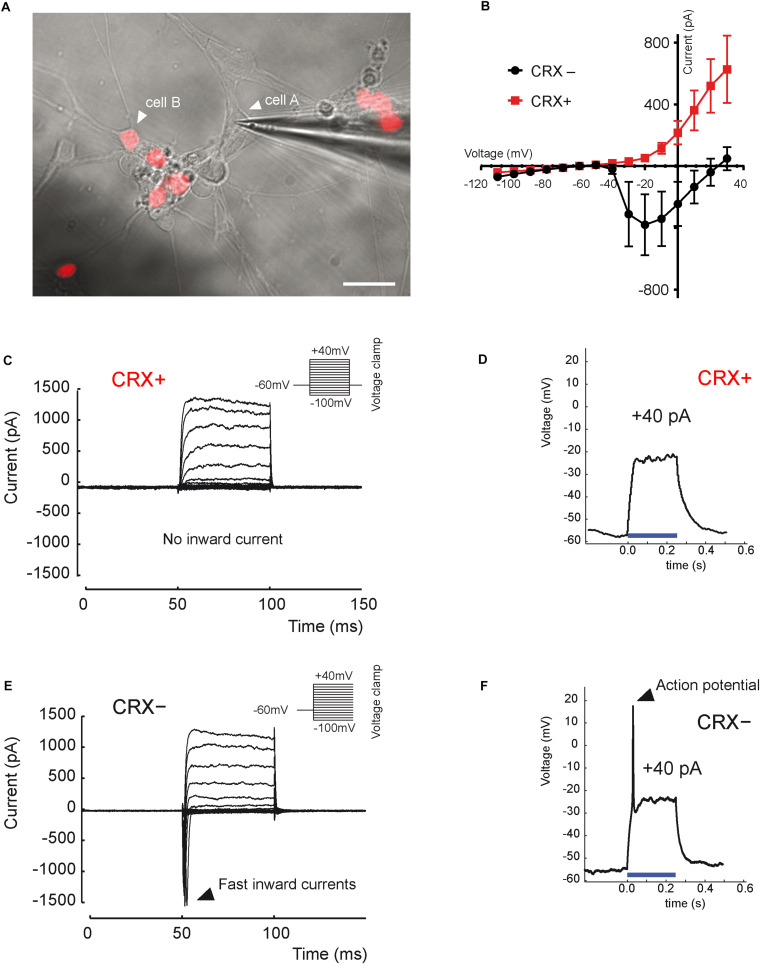
Distinct electrical properties of hiPSC-derived RGCs and hiPSC-derived CRX-positive photoreceptors. **(A)** Infrared image of a monolayer of retinal cells derived from D56 organoid generated from the adeno-associated virus integration site 1 (AAVS1):CrxP_H2BmCherry hiPSC line, 3 weeks after plating. Epifluorescence images revealing mCherry+ cells corresponding to CRX+ photoreceptors are superimposed (white arrow head on cell A; a recording electrode is in contact with a mCherry– (CRX–) non-photoreceptor cell showing an RGC morphology (white arrow head on cell A). Scale bar: 25 μm. **(B)** Mean current–voltage (I–V) relationship curve of CRX– (black curve) and CRX+ (red curve) cells, calculated as the minimum current value observed during the first 10 ms of each voltage step. Fast inward currents peaking around –20 mV are only visible for CRX– cells (mean ± SEM; *N* = 3; *n* = 7). **(C,E)** Current responses to voltage steps of CRX+ **(C)** and CRX– **(E)** representative cells by stepping the membrane potential from –100 to +40 mV in 10-mV increments (*N* = 3; *n* = 11). Only CRX– cells displayed fast inward currents. **(D,F)** Voltage responses of CRX+ **(D)** and CRX– **(F)** representative retinal cells to a +40-pA current injection (blue line). Evoked action potentials were observed only in CRX– cells (*N* = 3; *n* = 4).

Functional maturation of RGCs was further evaluated by single-cell electrophysiological recordings (Figure 3). Twenty out of sixty-one RGCs, defined by morphological criteria and recorded extracellularly showed spontaneous spiking (Figure 3A). The resting membrane potential was similar for all recording cells (−43.2 ± 2.1 mV; mean ± SEM), and the mean firing frequency was 0.31 ± 0.07 Hz (mean ± SEM; *n* = 20 out of 61 recorded cells from *N* = 9 differentiations). Voltage-gated sodium channels that mediate fast depolarization in neurons are responsible for initiation of the action potential (Marban et al., 1998). Patch-clamp recordings revealed fast inward ionic currents in a majority of putative RGCs (Figure 3B) (*n* = 15 out of 22 patched cells) and a voltage-dependence of these currents with an inward peak of activity at −20 mV. Because the majority of recorded cells were not spontaneously spiking, we injected positive current pulses during 500 ms leading to firing activity in the majority of recorded cells (Figure 3C) (*n* = 7 out of 10 cells). Firing response ranged from 1 to 10 action potentials per stimulation. Furthermore, in order to check if cells expressed functional glutamate receptors, we puffed glutamic acid (1 mM) and managed to record inward slow currents in response to the stimulation (Figure 3D) (*n* = 3 technical replicates). In order to better characterize the identity of recording cells, we performed similar experiments using our previously described CrxP_H2BmCherry reporter hiPSC line (Gagliardi et al., 2018). This allowed the identification of photoreceptors according to the expression of the mCherry fluorescent protein (Figure 4A). As expected, patch-clamp recordings showed that mCherry/CRX-positive photoreceptors failed to evoke fast inward currents (Figures 4B,C) (*n* = 7) and never evoked spike after current injection (Figure 4D) (*n* = 7). By contrast, mCherry/CRX-negative cells (classified as RGCs based on additional morphological criteria) displayed fast inward currents (Figures 4B,E) (*n* = 4) and evoked spikes after current injections (Figure 4F) (*n* = 3 out of 4). The resting membrane potential was similar for all recording cells, ranging from −38.3 ± 3.1 mV (mean ± SEM; CRX-positive cells) to −42.7 ± 4 mV (mean ± SEM; RGC-like CRX-negative cells). Based on these morphological and functional criteria, we demonstrated that RGCs isolated from retinal organoids can survive and mature in adherent mixed retinal cell cultures.

### Enrichment of RGCs by MACS Based on THY1 Expression in Adherent Cells From Dissociated Organoids

**FIGURE 5:**
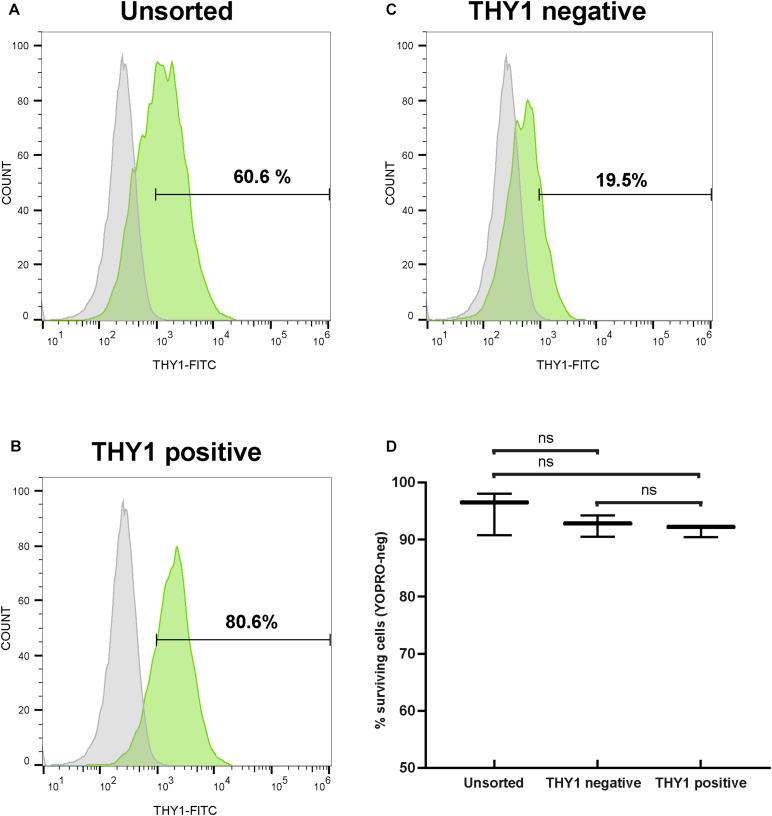
Selection of hiPSC-derived RGCs by targeting THY1. **(A–C)** One representative THY1 flow cytometry analysis plot of the different fractions before **(A)** and after magnetic-activated cell sorting (MACS) (**B:** THY1-positive fraction; **(C):** THY1-negative fraction). Fluorescence intensity indicative of FITC-conjugated THY1 primary antibody binding is shown on the *x*-axis. Specific staining in green; Non-specific binding was estimated by incubation with a mouse IgG1 FITC-conjugated isotypic antibody (in gray). **(D)** Quantitative analysis by flow cytometry of living cells (YOPRO-negative) on unsorted, THY1-negative, and THY1-positive fractions from retinal organoid-derived dissociated cells, 1 week after plating (min to max; *N* = 3 differentiations; *n* = 308 organoids; ns: not statistically significant two-way ANOVA followed by Tukey’s multiple comparison test).

Since the transplantation of hiPSC-derived RGCs represents one objective for future applications to treat optic neuropathies, we aimed to discard non-RGCs from the whole-cell population even though an enrichment in RGCs was already observed in our adherent cell culture conditions (Figure 2). For this purpose, 1 week after plating of dissociated retinal organoid cells, we performed MACS using THY1 antibody-coupled magnetic MicroBeads. In order to evaluate the enrichment of RGCs, unsorted and MAC-sorted fractions were submitted to flow cytometry to quantify THY1-positive cells. Flow cytometry analysis confirmed an RGC enrichment after 7 days in adherent cell culture conditions, with 60.48 ± 0.14% of THY1-positive cells in the unsorted fraction (Figure 5A) (mean ± SEM; *n* = 198 organoids; *N* = 3 independent experiments). We showed that the MACS-positive fraction containing 78.03 ± 1.47% of THY1-positive cells was significantly enriched compared to MACS-negative fraction containing 15.11 ± 3.77% of THY1-positive cells (Figures 5B,C) (mean ± SEM; *N* = 3; ^∗∗^*p* < 0.01; one-way ANOVA followed by Dunn’s multiple comparison test). Cell viability of MAC-sorted cells was assessed by flow cytometry using the apoptotic marker YOPRO (Figure 5D) showing a high survival rate in both THY1-positive and negative fractions (91.63 ± 0.62% and 92.5 ± 1.08%, respectively; *N* = 3; *n* = 308), similar to the living cell ratio before MACS (95.1 ± 2.19% in unsorted fraction; *N* = 3; two-way ANOVA).

**FIGURE 6:**
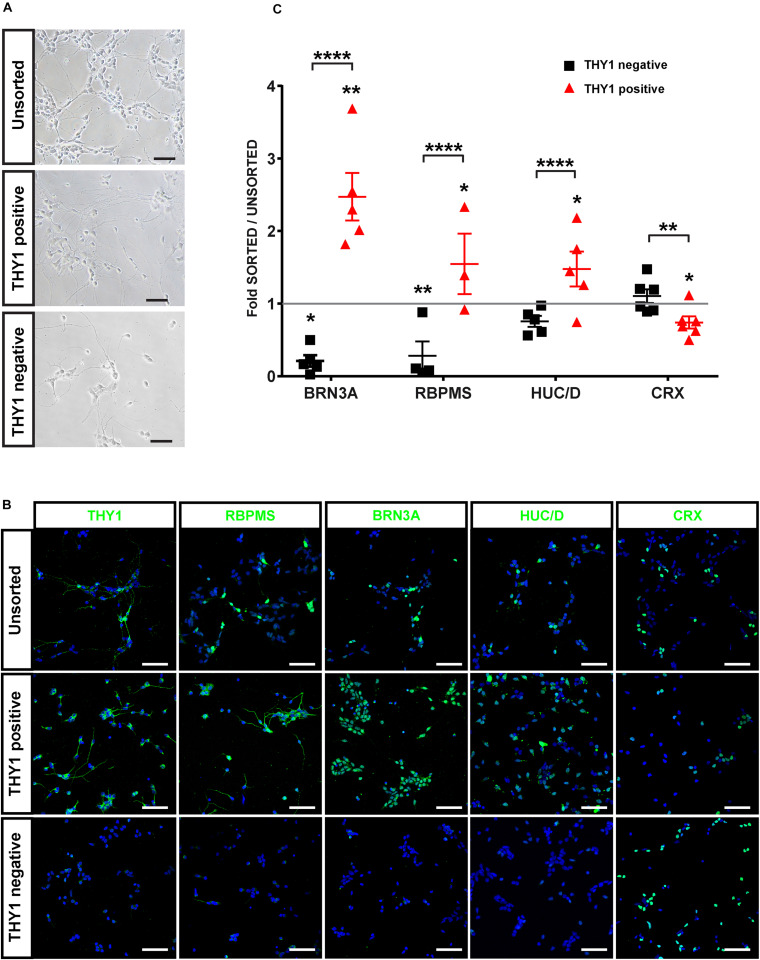
Characterization of hiPSC-derived RGCs after THY1-targeted MACS. **(A)** Phase-contrast and brightfield micrographs illustrate the morphology of dissociated cells from D56 retinal organoids 1 week after plating (unsorted) and 2 days after MACS (THY1-positive and THY1-negative fractions). Scale bars: 50 μm. **(B)** Immunofluorescence analysis of RGC markers (THY1, RBPMS, BRN3A, and HuC/D) and photoreceptor marker CRX in unsorted, THY1-positive, and THY-negative fractions (cell nuclei staining with DAPI in blue). Scale bars: 50 μm. **(C)** Quantitative analysis using ArrayScan of RGCs and photoreceptors in adherent cell cultures of unsorted, THY1-negative, and THY1-positive fractions, 2 days after plating (mean ± SEM; *p < 0.05, **p < 0.01, ****p < 0.0001; Dunn’s multiple comparisons test after global Kruskal–Wallis test; *N* ≥ 4). Asterisks positioned above scatter plots refer to comparison with the unsorted fraction (gray line); asterisks above square bracket refer to comparison between positive and negative fractions.

In order to confirm the identity of sorted cells (Figure 6A), we performed immunofluorescence analysis on cells from the different MACS fractions 2 days after plating (Figure 6B). As expected, the MACS-positive fraction was enriched in cells expressing specific RGC markers (THY1, BRN3A, RBPMS, HuC/D) (Figure 6B). Quantitative analysis revealed a significant enrichment in BRN3A-positive cells (+147% ± 38%) in the THY1-sorted cell population compared to the unsorted fraction and a significant depletion in the negative fraction (−80 ± 8%) (Figure 6C) (mean ± SEM; *n* = 671 organoids from *N* = 5 differentiations; ^∗∗^*p* < 0.01, Kruskal–Wallis test followed by Tukey’s multiple comparison test). This enrichment was confirmed by the increase in 55 ± 11.5% and 48 ± 7.7% of RBPMS-positive cells and HuC/D-positive cells, respectively, compared to the unsorted fraction (Figure 6C) (mean ± SEM; *N* = 5 experiments; ^****^p < 0.0001, Kruskal–Wallis test followed by Tukey’s multiple comparison test). In contrast, the MACS-positive fraction was partially depleted in CRX-positive photoreceptor cells (Figures 6B,C) (−24 ± 8.3% compared to the unsorted fraction; mean ± SEM; *N* = 6; *n* = 731 organoids; ^∗^*p* < 0.05, Kruskal–Wallis test followed by Tukey’s multiple comparison test). To demonstrate that this selection strategy was not dependent on the hiPSC line used for generation of retinal organoids, we carried out additional immunostaining with another previously characterized hiPSC line (hiPSC-2) derived from human dermal fibroblasts (Reichman et al., 2014). Using different markers of RGCs (THY1, HuC/D, and RBPMS), we confirmed an enrichment of RGCs in the THY1-sorted fraction (Supplementary Figure S2). In contrast, RCVRN-positive photoreceptors detected in the unsorted fraction were very rarely observed in the THY1-sorted fraction (Supplementary Figure S2).

**FIGURE 7:**
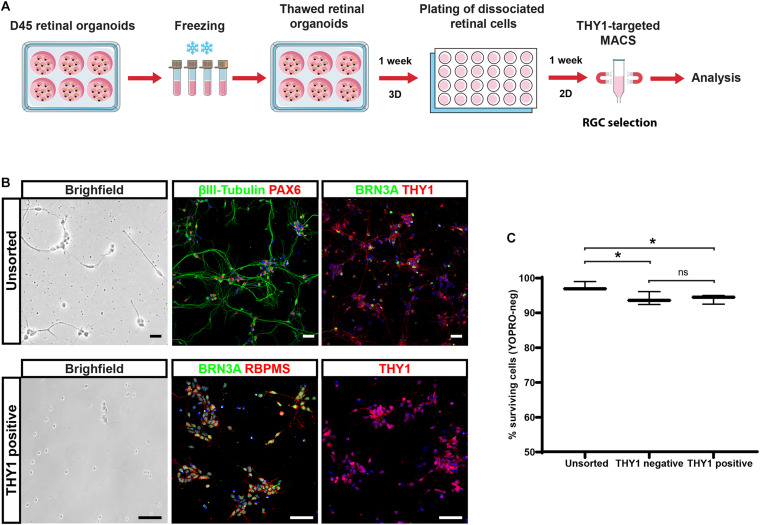
Selection of hiPSC-derived RGCs from cryopreserved retinal organoids. **(A)** Schematic diagram illustrating the protocol for the selection of THY1-targeted hiPSC-derived RGCs from cryopreserved hiPSC-derived retinal organoids. **(B)** Phase-contrast and brightfield micrograph and immunostaining for RGC markers, βIII-tubulin, BRN3A, PAX6, RBPMS, or THY1 in unsorted or THY1-sorted cells from dissociated freeze–thawed organoids (cell nuclei staining with DAPI in blue). Scale bars: 50 μm. **(C)** Quantitative analysis by flow cytometry of living cells (YOPRO negative) on unsorted, THY1-negative, and THY1-positive fractions, 1 week after plating, of dissociated cells derived from freeze–thawed retinal organoids (min to max; *N* = 3 differentiation; *n* = 380 organoids; *p < 0.05, two-way ANOVA followed by Tukey’s multiple comparison test).

With the perspective of developing cell therapy applications for optic neuropathies, we tested the ability to cryopreserve dissociated cells before or after THY1-targeted MACS. Dissociated cells before cell sorting (unsorted fraction) and MAC-sorted cells were immediately frozen, thawed 1 week later, and cell viability was assessed by flow cytometry using the apoptotic marker YOPRO. Quantitative analysis demonstrated an important decrease in cell viability in freeze–thawed cell population, both in unsorted (51.4 ± 5.2%; *N* = 3) and THY1-sorted (24.5 ± 0.5%; *N* = 3) fractions compared to corresponding fresh-cell fractions: 99.4 ± 0.3% and 94.5 ± 1.2% for unsorted and THY1-sorted fraction, respectively (*n* = 395 organoids from *N* = 3 experiments; ^****^p < 0.0001, two-way ANOVA followed by Tukey’s multiple comparison test). These results ruled out the possibility to use this approach for banking THY1-targeted MACS retinal cells. Having already validated the possibility to cryopreserve retinal organoids (around D85-D100) without affecting photoreceptor differentiation after thawing (Gagliardi et al., 2018), we tested the possibility, as an alternative solution, to isolate RGCs from young freeze–thawed organoids (Figure 7). Retinal organoids frozen at D45 of differentiation were thawed and put back into floating culture for 1 week before dissociation. Retinal cells were then cultured an additional week in adherent conditions (Figure 7A). Immunostaining demonstrated that most cells exhibited an RGC profile with the expression of specific neuronal, retinal, and RGC markers such as βIII-tubulin, PAX6, BRN3A, or THY1 (Figure 7B). In these conditions, THY1-targeted MACS was still efficient as demonstrated by immunostaining on the sorted fraction in which a majority of the cells expressed BRN3A, RBPMS, and THY1 (Figure 7B). One week after dissociation, quantitative analysis of YOPRO labeling by flow cytometry demonstrated that freeze–thawing of retinal organoids did not affect cell viability in dissociated cells (unsorted cells) from freeze–thawed organoids (97.43 ± 0.74%; *N* = 3) (Figure 7C) compared to fresh organoids (95.1 ± 2.19; *N* = 3) (Figure 5D). A high survival rate was also observed after MACS (Figure 7C) (93.90 ± 0.76% in positive fraction and 93.93 ± 1.09% in negative fraction; *N* = 3) even though cell viability was slightly lower compared to unsorted cells (*n* = 380 organoids; *N* = 3 experiments; ^∗^*p* < 0.05, two-way ANOVA followed by Tukey’s multiple comparison test). These results suggested that performing THY1-targeted MACS on retinal dissociated cells derived from previously cryopreserved organoids could provide a good solution to obtain readily transplantable cells and overcome the obstacle of direct cryopreservation of THY1-targeted MACS retinal cells.

### Generation and Retinal Differentiation of Human Reporter AAVS1::CAG-P_EGFP iPSC Line

**FIGURE 8:**
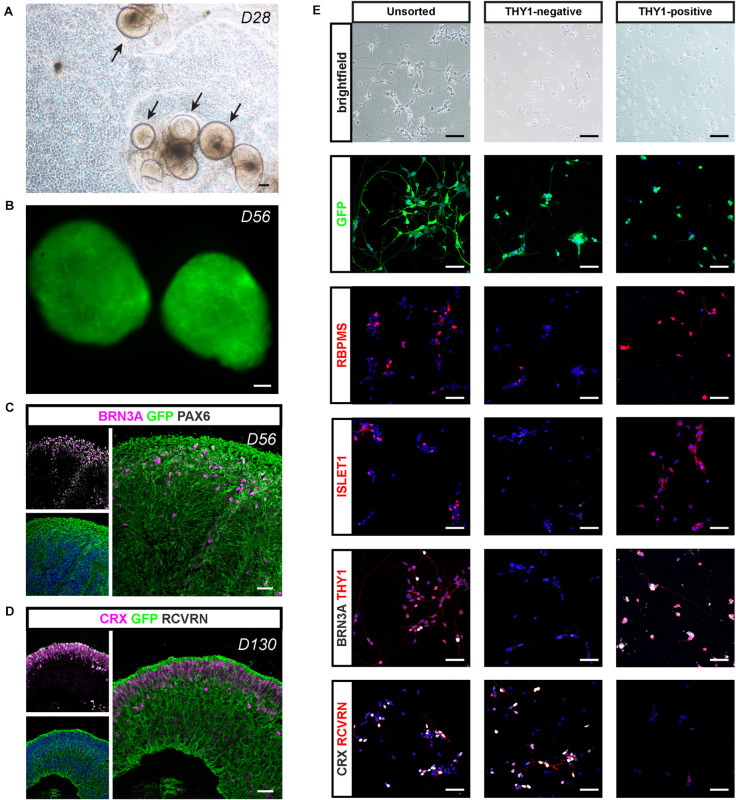
Retinal differentiation and selection of THY1-positive RGCs using a AAVS1:CAG-P_EGFP hiPSC line. **(A)** Phase-contrast and brightfield micrograph showing emergence of D28 retinal organoids from differentiating AAVS1:CAG-P_EGFP hiPSCs. Scale bar: 200 μm. **(B)** Endogenous GFP expression in D56 retinal organoids. Scale bar: 200 μm. **(C,D)** Immunostaining showing the expression of GFP in all retinal organoid cells. **(C)** RGCs are identified at D56 according to the co-expression of PAX6 and BRN3A. **(D)** Co-expression of CRX and recoverin (RCVRN) corresponds to photoreceptors in D130 organoids. Scale bars: 30 μm. **(E)** RGC characterization in unsorted, THY1– and THY1+ fractions according to the expression of RBPMS, ISLET1, BRN3A, and THY1. Immunostaining for CRX and RCVRN enables photoreceptor identification. Scale bars: 50 μm.

For potential validation of cell replacement strategies, we engineered a fluorescent reporter human iPSC line (Supplementary Figure S3) from hiPSC line-5f (Slembrouck-Brec et al., 2019) enabling the production of fluorescent retinal cells that could be easily identified after transplantation. We used the CRISPR-Cas9 knock-in strategy (Cong et al., 2013; Gagliardi et al., 2018) to generate an hiPSC reporter line in which the fluorescent protein EGFP under the control of ubiquitous CAG promoter is inserted into the “safe harbor” AAVS1 site (Supplementary Figure S3A). Among the five puromycin-resistant selected clones (Supplementary Figure S3B), we selected a clone carrying a copy of the insert in both AAVS1 loci (CAG-c2), for further retinal differentiation. The iPSC line genomic integrity was confirmed by SNP genotyping (Supplementary Figure S3C). As expected, the iPSC colonies expressed GFP and the pluripotency markers OCT4, SSEA4, SOX2, and NANOG (Supplementary Figures S3D,E). When overgrowing AAVS1:CAG-P-EGFP iPSCs were switched to a differentiation medium, self-forming neuro-retinal structures could be observed 4 weeks after the initiation of differentiation (Figure 8A). Endogenous GFP signal was visible in D56 retinal organoids in floating cultures (Figure 8B). Immunostaining for GFP on cryosections of retinal organoids confirmed the expression of the transgene in all retinal cells (Figures 8C,D). Co-immunostaining with specific RGC markers (BRN3A and PAX6) in D56 organoids (Figure 8C) or photoreceptor markers (CRX and RCVRN) in D130 organoids (Figure 8D) confirmed that GFP is expressed in differentiating retinal neurons.

Applying the double-stepwise selection of RGCs (adherent culture of dissociated cells from D56 retinal organoids combined with THY1-targeted MACS) to GFP-positive retinal organoids, allowed an enrichment in cells expressing RGC markers (RBPMS, BRN3A, THY1, and ISLET1) in the MACS-positive fraction compared to unsorted cells and MACS-negative fraction (Figure 8E). As expected, the MACS-positive fraction was markedly depleted in photoreceptor cells identified with both CRX and RCVRN antibodies, while they were mainly found in the MACS-negative fraction (Figure 8E). These results confirm that our optimized protocol for RGC generation and enrichment from human retinal organoids is suitable for different hiPSC lines.

### Transplantation of Unsorted and THY1-Positive Retinal Cells Into a Mouse Model of Optic Nerve Injury

**FIGURE 9:**
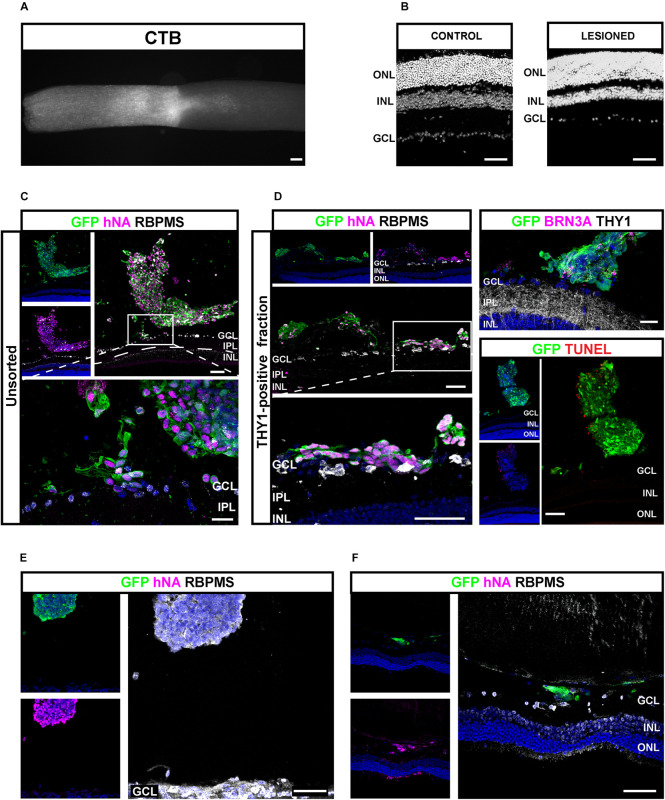
Injection of hiPSC-derived RGCs in the vitreous of optic nerve crush mice model. **(A)** Alexa 555-conjugated CTB-β-labeled optic nerve crush revealing the lesion site. Scale bar: 100 μm. **(B)** DAPI staining showing the RGC loss in the GCL 5 weeks after optic nerve crush. Scale bar: 100 μm. **(C–F)** Immunostaining in retinal sections from mice grafted with unsorted **(C)** or MAC-sorted THY1-positive fractions **(D)** 1 week after transplantations, and MAC-sorted THY1-positive cells **(E,F)** 4 weeks after transplantations. The delimitated areas indicate the location of high-magnification image below. Transplanted cells are identified according to GFP and human marker hNA immunoreactivity **(C–F)**. RGCs are identified according to BRN3A **(D)** or RBPMS expression **(C–F)**. Notice the close apposition of transplanted cells to GCL **(D)**. Evaluation of cell death among engrafted cells was performed according to TUNEL labeling **(D)**. Notice the small number of TUNEL-positive cells. Scale bars: 100 μm **(A–D)** or 50 μm **(E,F)**. GCL, ganglion cell layer; INL, inner nuclear layer; IPL, inner plexiform layer; ONL, outer nuclear layer.

Next, we sought to study the transplantation competence of hiPSC-derived RGCs in a host environment recapitulating degenerative conditions of RGCs. Progressive loss of optic nerve fibers was induced by the optic nerve crush (ONC) model (Park et al., 2008; McKinnon et al., 2009). ONC was confirmed after tracing RGC axons through intravitreal injection of Alexa-555-conjugated cholera-toxin subunit B (CTB) (Figure 9A). ONC was followed by a progressive loss of endogenous RGCs visualized after 4 weeks (Figure 9B). Unsorted cells (200,000) (Figure 9C) or THY1-sorted cells (Figures 9D–F) derived from GFP-expressing retinal organoids were injected into the vitreous space close to the host ganglion cell layer (GCL) 4 weeks after ONC. One week after transplantation in immunosuppressed animals, GFP-positive cells were found in 75% of eyes injected with unsorted cells (*n* = 3 out of 4) and 59% of eyes injected with THY1-sorted cells (*n* = 10 out of 17 mice; *N* = 3 experiments). GFP-positive injected cells in the vitreous were clearly identified by the expression of human-specific marker hNA (Figures 9C,D). Most human grafted GFP-positive cells co-expressed RBPMS (Figures 9C,D), and only some expressed BRN3A and THY1 (Figure 9D and Supplementary Figure S4) in accordance with the previous demonstration that BRN3A is expressed in an RGC subpopulation (Badea et al., 2009). In order to exclude the possibility that injected cells engaged in apoptosis, we performed TUNEL assay (Figure 9D). Quantification of TUNEL-positive cells revealed that 15.82 ± 4.98% of GFP-positive cells were TUNEL positive demonstrating that most grafted cells were alive (Figure 9D) (*n* = 9). Among the animals injected with THY1-sorted cells and displaying human cells in the vitreous, nine showed GFP-positive cells intermingled with the host GCL. In these animals, some double-positive RBPMS/GFP-injected RGCs migrated in the remaining GCL (Figures 9C,D). Interestingly, GFP/hNA double-positive cells were still present 4 weeks after injection of THY1-sorted cells in half of the transplanted animals (*n* = 3 out of 6) under immunosuppressive treatment. Transplanted cells were distributed either as small cell clusters close to the retina (Figure 9E) or as small number of cells, intermingled with the host GCL (Figure 9F). Despite no evidence of neurite outgrowth, the majority of surviving cells were identified as RGCs according to RBPMS expression (Figures 9E,F).

## Discussion

In the current study, we have developed a two-step process to optimize the differentiation and the isolation of RGCs from hiPSC-derived retinal organoids. As previously reported in different retinal organoids (Reichman et al., 2014; Zhong et al., 2014), we demonstrated an increase in the RGC population from D42 with a peak around D56 before a progressive loss of these cells. The RGC loss starting before their advanced maturation, as shown by the absence of THY1 immunoreactivity, was likely due to the absence of projection targets and to the floating culture condition, the latter representing a strong hindrance to axon outgrowth outside the organoids, as previously observed with an RGC fluorescent reporter iPSC line (Capowski et al., 2019). Beyond the morphological and molecular features of RGCs, the functional maturation, such as the ability of RGCs to trigger action potentials, is an important issue. By using MEA recording, we detected relatively low spontaneous activity, which does not exclude that more RGCs would be able to fire in response to stimulation. Moreover, scattered distribution of RGCs in the core of retinal organoids may limit suitable contacts with the recording electrodes required for an optimal recording. Despite these technical issues, the possibility to detect spiking activity in retinal organoids suggests that RGCs initiated a maturation process. RGCs are massively predominant among the spiking retinal cells. However, it cannot be totally excluded that some other retinal cells, such as a few amacrine cells, are at the origin of some recorded spikes.

In order to promote RGC survival and functional maturation, retinal organoids have been dissociated at D56, a stage of development at which many RGCs are present, and retinal cells were replated onto an adherent substrate. In agreement with previous studies, the adherent culture of retinal cells allowed RGCs to grow extensions showing βIII-tubulin or THY1 immunoreactivity (Ohlemacher et al., 2016; Li et al., 2017; Fligor et al., 2018; Langer et al., 2018; Chavali et al., 2020). The capacity of neurons such as RGCs to trigger an action potential depends on different hallmarks including the expression of specific voltage-gated channels and, importantly, a resting membrane potential (V_rest_) allowing their activation (Catterall, 2012). This crucial point is illustrated by a recent study showing that only hiPSC-derived RGCs displaying an adequately negative V_rest_ were able to spike (Teotia et al., 2019). In the present study, the V_rest_ was approximatively −43 mV, a slightly more depolarized value than that reported by Riazifar et al. (2014) or Chavali et al. (2020) in hiPSC-derived RGC-like cells, but lower than that observed by Teotia et al. (2019). Consistent with previous studies (Teotia et al., 2019; VanderWall et al., 2019), we demonstrated that 3 weeks after plating, the mature RGCs, in contrast to photoreceptors, displayed voltage-gated ionic currents enabling these RGCs to evoke spikes after stimulation and to spontaneously fire. The ability of RGCs to trigger action potentials is an important issue for future transplantation applications since electrical activity has been shown to be important for both RGC development (Assali et al., 2014) and regeneration (Lim et al., 2016). Interestingly, MAC-sorted RGCs displayed very weak voltage-gated fast inward currents that were insufficient to allow the generation of an action potential (data not shown), suggesting that functional maturation of RGCs could be improved in the presence of other non-RGC retinal cells. Similar observations have been nicely reported for hiPSC-derived RGCs co-cultured with astrocytes by VanderWall et al. (2019).

The cell sorting method based on the specific expression of a cell surface marker is useful for the enrichment of a specific cell type, allowing for instance, transcriptomic analysis (Daniszewski et al., 2018). In the present study, we demonstrated that adherent culture conditions of dissociated cells from D56 retinal organoids are sufficient to enrich in RGCs more than 60%, according to THY1 expression, 1 week after plating. The second step of RGC enrichment using THY1-targeted MACS allowed to reach up to almost 80% of RGCs, corresponding to a similar enrichment than previously reported with another RGC differentiation protocol (Gill et al., 2016). To improve MACS efficiency, retinal cell culture after plating could be extended in order to obtain more mature RGCs expressing a higher level of THY1. However, for future cell transplantation, the ontogenetic stage of donor cells should be a critical point. The ability of RGCs to integrate the host tissue according to the specific developmental stage is poorly documented, but it was shown in rodents that RGCs isolated from adult retina failed to integrate the host retina after intravitreal injection unlike RGCs isolated from early postnatal donors (Hertz et al., 2014; Venugopalan et al., 2016). An excessive stage of differentiation of engrafted cells could also explain integration failure observed in normal mouse retina with RGC-like cells derived from mouse iPSC overexpressing Atoh7 in order to bias RGC differentiation toward RGC lineage (Chen et al., 2010). In order to efficiently select RGCs, other strategies using engineered reporter cell line expressing fluorescent protein or a cell surface selection marker have been proposed (Sluch et al., 2015, 2017). However, engineered reporter cell lines may not be suitable for the development of clinically compatible stem cell-based therapies.

One major advantage of the separation strategy presented here relies on the possible transfer of our entire protocol to GMP-compatible conditions. Indeed, our differentiation protocol is based on a feeder-free iPSC culture system and uses exclusively chemically defined xeno-free compounds. Furthermore, the GMP-compliant MACS enrichment is already used in clinical trials (Menasché et al., 2015). We also demonstrated that MACS targeting THY1 expression could be performed using cryopreserved organoids, ensuring the possibility to store the organoids at the appropriate stage of differentiation for downstream applications. Targeting other specific RGC cell surface markers such as CD184 or CD171 (Aparicio et al., 2017) could be an interesting alternative to improve RGC enrichment compared to THY1 targeting.

Transplantation studies demonstrated that THY1-enriched retinal cells survived in the vitreous up to 4 weeks after injection. Cells were found in the eyes of 59% of transplanted animals 1 week after intravitreal injection and 50% of transplanted animals 4 weeks post-injection. The limited efficacy of the immunosuppressive regimen could explain the limited number of animals where human cells can be detected, even though we cannot formally rule out some technical issues related to intravitreal injections. Administration of an immunosuppressant in drinking water is classically used and has demonstrated its efficiency (Jensen et al., 2012; Kamao et al., 2014; Ben M’Barek et al., 2017) but can yield partial success because of the inconsistent blood immunosuppressant levels (Jensen et al., 2012). However, the success ratio of transplantation is comparable and even slightly higher to the one (43%) reported with intravitreal injection of postnatal rat RGCs in normal mice (Venugopalan et al., 2016). TUNEL experiments demonstrated that the majority of injected cells were alive and immunostaining with specific RGC markers and specific antibodies for human antigen confirmed that most of the injected human cells were RGCs. Many engrafted cells were observed as trapped in the vitreous. Nevertheless, in a few animals, the engrafted cells intermingled with host retina. Although further experiments are needed to clearly demonstrate their integration into the host retina, our data are consistent with previous studies reporting 10% integration efficiency of postnatal rat RGCs (Venugopalan et al., 2016). A recent study suggested that retinal progenitors derived from human embryonic stem cells (hESCs) could differentiate into RGC-like cells in the host GCL 4 weeks after transplantation in NMDA-injured retina, but no morphological maturation was reported (Wang et al., 2019). However, injection of a cell population that may contain mitotic cells seems hazardous, with the risk of cell hyperproliferation within the vitreous. Very recently, intravitreal injection of hESC-derived RGC-like cells has been performed in normal, non-injured adult rat eye showing some survival and migration of transplanted cells into the host GCL (Zhang et al., 2019). Some donor cells displayed β-III-tubulin labeling suggesting initiation of maturation, but according to the authors, donor cells failed to express specific RGC marker such as RBPMS. In our study, transplanted cells did not harbor neurite elongation, even at 4 weeks after transplantation, in contrast to the observation made by Venugopalan et al. (2016) with transplantation of rat postnatal RGCs. These authors reported that 3 weeks after transplantation, some engrafted cells harbored neurite elongation on the retinal surface with diverse dendrite architecture demonstrating that RGC could morphologically mature after transplantation. Slower kinetics of human development could explain that transplanted cells failed to harbor significant neurite outgrowth, and increasing the delay after transplantation of hiPSC-derived RGCs would be useful to explore the ability of RGCs to extend axons and dendrites and to establish synaptic contacts with bipolar and amacrine cells in our model of optic nerve injury. RGC scattering in the vitreous after injection has been frequently reported (Hertz et al., 2014; Becker et al., 2016). Co-injection with trophic factors supporting axon growth would be also useful. Interestingly, hiPSC-derived RGCs have been shown to be responsive to various factors such as BDNF (Fligor et al., 2018). In order to facilitate cell migration in the vitreous to reach the retinal surface, it would be useful to limit the aggregation of transplanted cells with the use of specific injectable hydrogels (Pakulska et al., 2012). The inner limiting membrane, and more generally the extracellular matrix, could also limit cell integration into the GCL layer. Co-injection with a specific enzyme, such as chondroitinase, should facilitate both migration and integration of transplanted cells, as it has been reported for the injection of Muller glial cell-derived RGC-like cells (Singhal et al., 2012).

Attachment of the grafted cells onto the host retina surface is also a critical point. The absence of physical support possibly limits the survival and differentiation of the injected cells. Some recent studies demonstrated that engineered scaffolds or biomaterials can stimulate RGC axon outgrowth *in vitro* (Kador et al., 2014; Sluch et al., 2015; Li et al., 2017; Yang et al., 2017). Introducing a RGC-scaffold biomaterial into the eye would be a real progress compared to an injection of cell suspension as reported recently in rabbits and monkeys (Li et al., 2017). In summary, we report the optimization of RGC generation derived from hPSCs compatible with GMP manufacturing required for future cell replacement therapies. To the best of our knowledge, this is the first “proof of concept” of a successful transplantation of isolated hPSC-derived RGCs in a mouse model of optic neuropathy.

## Data Availability Statement

The data used to support the findings of this study are available from the corresponding author upon request.

## Ethics Statement

The animal study was reviewed and approved by the Charles Darwin Ethical Committee for Animal Experimentation C2EA-05.

## Author Contributions

OR conducted the conception and design, collection and assembly of data, data analysis and interpretation, manuscript writing, and final approval of the manuscript. ACha carried out the collection and assembly of data, data analysis and interpretation, and final approval of the manuscript. AM was carried out the collection and assembly of data, data analysis, and interpretation. CJ was involved in the collection and assembly of data. AS-B, CN, and AR were contributed to the provision of study materials. GG and SR were involved in the provision of study materials and data analysis and interpretation. J-AS contributed to administrative and financial support. JD contributed to data analysis and interpretation. AChe contributed to the data analysis and interpretation, and final approval of the manuscript. OG was involved in the conception and design, data analysis and interpretation, manuscript writing and final approval of the manuscript, and financial support. GO was responsible for the conception and design, collection and assembly of data, data analysis and interpretation, manuscript writing and final approval of the manuscript, and financial support. All authors contributed to the article and approved the submitted version.

## Conflict of Interest

The authors declare that the research was conducted in the absence of any commercial or financial relationships that could be construed as a potential conflict of interest.
