# Supplementary material for: Generation of a Transplantable Population of Human iPSC-Derived Retinal Ganglion Cells
Source: Front Cell Dev Biol. 2020 Oct 27;8:585675. doi: 10.3389/fcell.2020.585675 (PMC7652757; doi:10.3389/fcell.2020.585675)
Supplement: Supplementary file 1 [file Table_1.docx]

### Supplemental Table 1: list of primary and secondary antibodies

| **Antigen** | **Species** | **Dilution** | **Source** |
| --- | --- | --- | --- |
| BRN3A | Mouse monoclonal | 1:250 | Millipore (MAB1585) |
| CRX | Mouse monoclonal | 1:5000 | Abnova (H00001406-M02) |
| GFP | Chicken polyclonal | 1:200 | Aves Labs (GFP-1010) |
| hNUCLEI | Mouse monoclonal | 1:500 | Millipore (MAB1281) |
| HuC/D | Mouse monoclonal | 1:500 | ThermoFisher Scientific (AF21271) |
| ISLET1 | Rabbit polyclonal | 1:1000 | Abcam (ab20670) |
| NANOG | Rabbit monoclonal | 1:200 | Cell signalling (#4903) |
| OCT4 | Rabbit monoclonal | 1:100 | Cell signalling (#2840) |
| PAX6 | Rabbit polyclonal | 1:2000 | Millipore (AB2237) |
| PAX6 | Mouse monoclonal | 1:1000 | DSHB (AB_528427) |
| RBPMS | Rabbit polyclonal | 1:600 | Phosphosolutions (1830-RBPMS) |
| RBPMS | Mouse monoclonal | 1:600 | Abcam (ab128081) |
| RECOVERIN | Rabbit polyclonal | 1:2000 | Millipore (AB5585) |
| SOX2 | Rabbit monoclonal | 1:400 | Cell signalling (#3579) |
| SSEA4 | Mouse monoclonal | 1:200 | Cell signalling (#4755) |
| THY1 (CD90) | Rabbit monoclonal | 1:650 | Abcam (ab133350) |
| THY1 (CD90)-FITC | Mouse monoclonal | 1 : 200 | eBio5E10, eBioscience (11-0909-41) |
| TUBB3 (β3-tubulin) | Mouse monoclonal | 1:1000 | Covance (#MMS-435P) |
| Alexa fluor 488 anti-chicken | Donkey | 1:800 | Jackson ImmunoResearch 703-545-155 |
| Alexa fluor 488 anti-goat | Donkey | 1:800 | Thermofisher Scientific A-11055 |
| Alexa fluor 488 anti-mouse | Donkey | 1:800 | Jackson ImmunoResearch 715-545-150 |
| Alexa fluor 488 anti-rabbit | Donkey | 1:800 | Jackson ImmunoResearch 711-545-152 |
| Alexa fluor 594 anti-mouse | Donkey | 1:800 | Jackson ImmunoResearch 715-585-150 |
| Alexa fluor 594 anti-rabbit | Donkey | 1:800 | Jackson ImmunoResearch 711-585-152 |
| Alexa fluor 647 anti-mouse | Donkey | 1:800 | Jackson ImmunoResearch 715-605-150 |
| Alexa fluor 647 anti-rabbit | Donkey | 1:800 | Jackson ImmunoResearch 711-605-152 |
