# Supplementary material for: Generation of a Transplantable Population of Human iPSC-Derived Retinal Ganglion Cells
Source: Front Cell Dev Biol. 2020 Oct 27;8:585675. doi: 10.3389/fcell.2020.585675 (PMC7652757; doi:10.3389/fcell.2020.585675)
Supplement: Supplementary file 2 [file Table_2.docx]

### Supplemental Table 2: List of TaqMan® Gene Expression ID Assays used for qRT-PCR

| **Gene** | **Assays ID** |
| --- | --- |
| THY1 | Hs00264235_s1; THY1 |
| BRN3A / POU4F1 | Hs0036671_m1; POU4F1 |
| BRN3B / POU4F2 | Hs00231820_m1; POU4F2 |
| RBPMS | Hs01596836_m1; RBPMS |
| 18S | Hs99999901_s1 ; 18S |
