# Supplementary material for: Generation of a Transplantable Population of Human iPSC-Derived Retinal Ganglion Cells
Source: Front Cell Dev Biol. 2020 Oct 27;8:585675. doi: 10.3389/fcell.2020.585675 (PMC7652757; doi:10.3389/fcell.2020.585675)
Supplement: Supplementary file 3 [file Image_1.PDF]

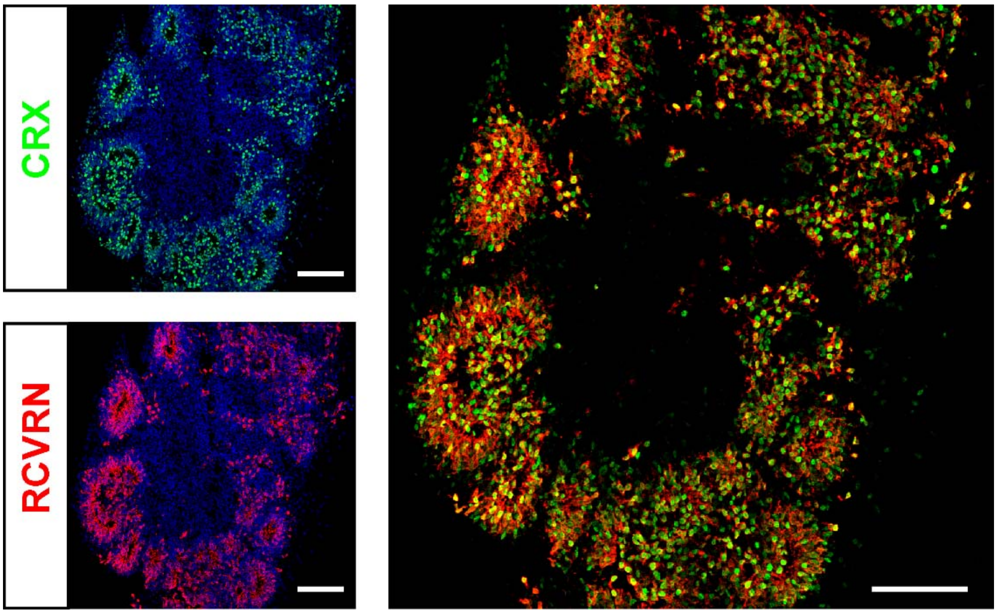

Supplementary Figure S1. Photoreceptor population in hiPSC-derived retinal organoids
Immunostaining showing the expression of CRX and Recoverin (RCVRN) in sections of D98 retinal organoids identifying photoreceptors. Nuclei were counterstained with DAPI (blue). Scale bars, 100 µm.
