# Supplementary material for: Generation of a Transplantable Population of Human iPSC-Derived Retinal Ganglion Cells
Source: Front Cell Dev Biol. 2020 Oct 27;8:585675. doi: 10.3389/fcell.2020.585675 (PMC7652757; doi:10.3389/fcell.2020.585675)
Supplement: Supplementary file 4 [file Image_2.PDF]

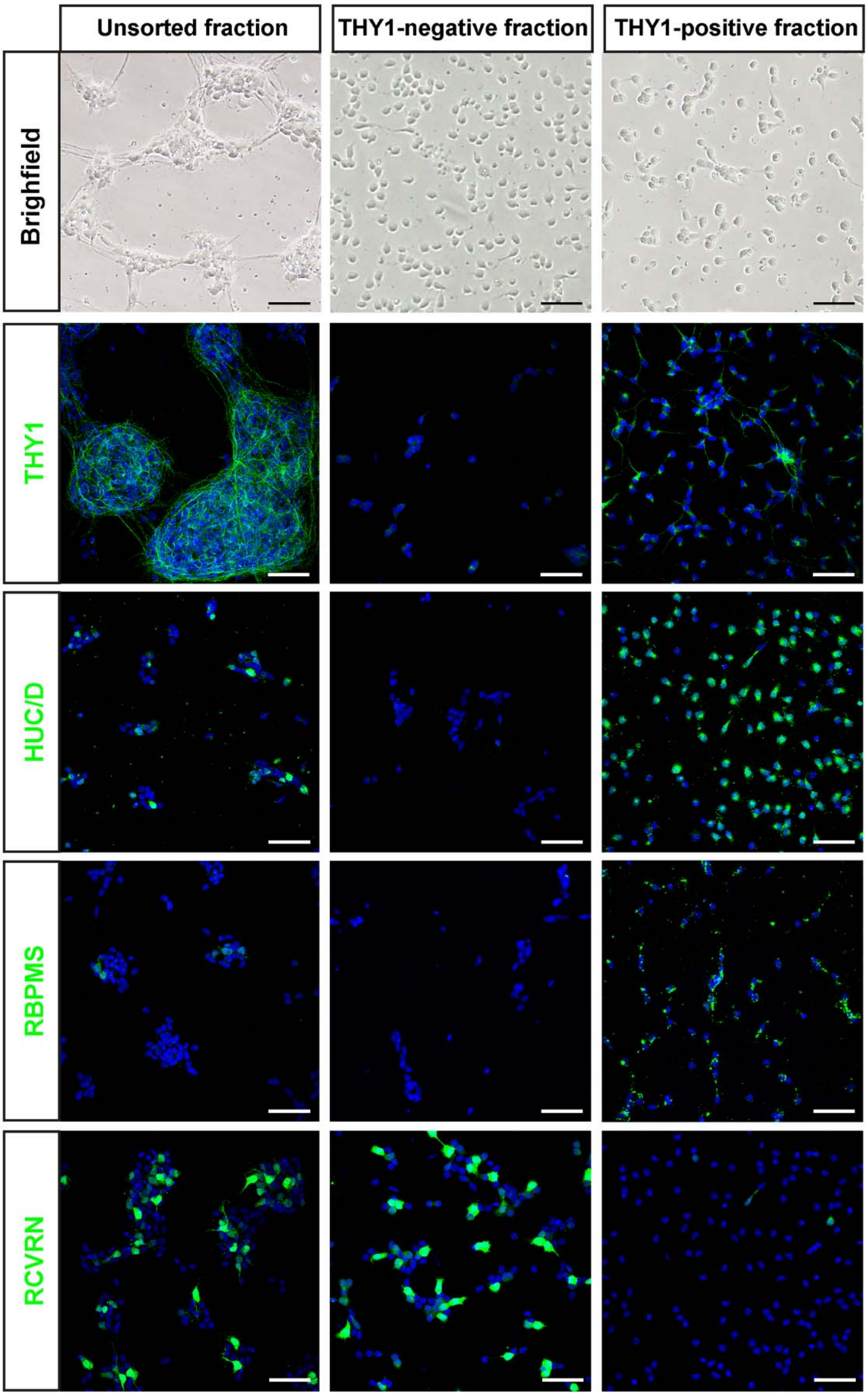

Supplementary Figure S2. Reproducibility of generation of retinal organoids and selection of hiPSC-derived RGCs with an additional hiPSC line (hiPSC-2).

Phase-contrast and brightfield micrographs and immunofluorescence analysis showing the expression of RGC markers (THY1, HuC/D and RBPMS) and photoreceptor markers (RCVRN) in unsorted, THY1-negative and THY-positive cell fractions. Scale bars, 50µm.
