# Supplementary material for: Generation of a Transplantable Population of Human iPSC-Derived Retinal Ganglion Cells
Source: Front Cell Dev Biol. 2020 Oct 27;8:585675. doi: 10.3389/fcell.2020.585675 (PMC7652757; doi:10.3389/fcell.2020.585675)
Supplement: Supplementary file 5 [file Image_3.PDF]

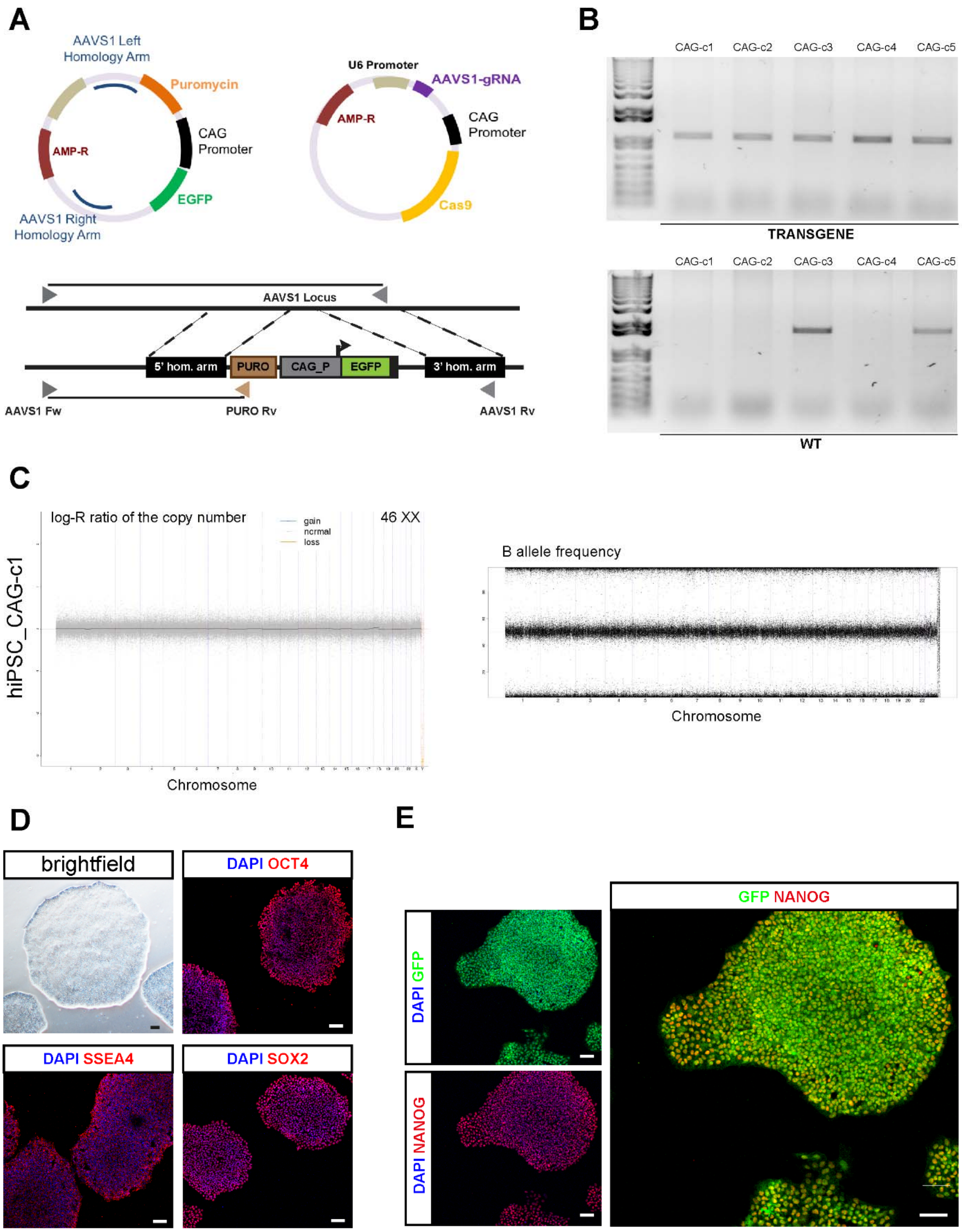
A
AAVS1 Left Homology Arm
Puromycin
CAG Promoter
AMP-R
EGFP
AAVS1 Right Homology Arm
U6 Promoter
AAVS1-gRNA
AMP-R
CAG Promoter
Cas9
AAVS1 Locus
5' hom. arm
PURO
CAG_P
EGFP
3' hom. arm
AAVS1 Fw
PURO Rv
AAVS1 Rv
B
CAG-c1
CAG-c2
CAG-c3
CAG-c4
CAG-c5
TRANSGENE
WT
C
hiPSC_CAG-c1
log-R ratio of the copy number
gain
normal
loss
46 XX
Chromosome
B allele frequency
Chromosome
D
brightfield
DAPI OCT4
DAPI SSEA4
DAPI SOX2
E
DAPI GFP
DAPI NANOG
GFP NANOG

Supplementary Figure S3. Generation of a fluorescent reporter AAVS1::CAG-P_EGFP iPSC line.

(A) Schematic representation of CRISPR/Cas9 and donor plasmids used for the generation of the AAVS1::CAG-P_EGFP iPSC line. Triangles on the schema indicate the homology region of the primers (AAVS1 Fw, PURO Rv, AAVS1 Rv) used to validate the insertion of the fluorescent cassette.

(B) Results of the PCR evaluating the integration of CAG-P_EGFP construct into AAVS1 site. Gel on the top shows the presence of the transgene in all puromycin-selected clones corresponding to the specific amplification of the inserted product with AAVS1 Fw (upstream the integration site) and PURO Rv primers (within the cassette), as indicated in A. Gel on the bottom shows the result of the PCR with AAVS1 Fw and AAVS1 Rv primers (in the AAVS1 right homologous arm) amplifying exclusively the WT form of the AAVS1 locus allowing for distinction between homozygous (CAG-c1, CAG-c2 and CAG-c4) or heterozygous (CAG-c3 and CAG-c5) integration.

(C) SNP array analysis of reporter iPSC line CAG-c2 8 passages after puromycin selection. Data are presented as the weighted log2 ratio of the copy number (Y-axis on left panels) or B allele frequency (Y-axis on right panels) and the chromosome number (X-axis).

(D, E) Immunohistochemistry of pluripotency markers (SSEA4, OCT4, SOX2 and NANOG) and GFP on iPSC line CAG-c2 five passages after puromycin selection. Scale bars, 100 µm.
