# Supplementary material for: Generation of a Transplantable Population of Human iPSC-Derived Retinal Ganglion Cells
Source: Front Cell Dev Biol. 2020 Oct 27;8:585675. doi: 10.3389/fcell.2020.585675 (PMC7652757; doi:10.3389/fcell.2020.585675)
Supplement: Supplementary file 6 [file Image_4.PDF]

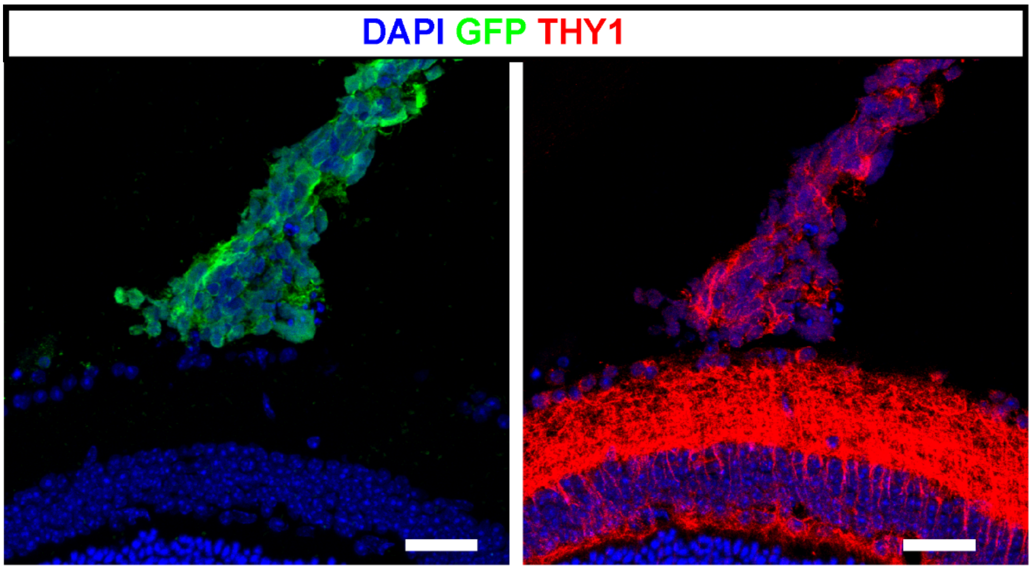

Supplementary Figure S4. hiPSC-derived THY1-GFP double positive transplanted cells

Immunostaining in retinal section from mice grafted with MAC-sorted THY1-positive fractions showing GFP-THY1 double positive cells close to the ganglion cell layer of the host retina. Nuclei were counterstained with DAPI (blue). Scale bars, 30 µm.
